# Detection of Novel Goose Parvovirus Disease Associated with Short Beak and Dwarfism Syndrome in Commercial Ducks

**DOI:** 10.3390/ani10101833

**Published:** 2020-10-08

**Authors:** Mohamed A. Soliman, Ahmed M. Erfan, Mohamed Samy, Osama Mahana, Soad A. Nasef

**Affiliations:** Reference Laboratory for Veterinary Quality Control on Poultry Production, Animal Health Research Institute, Agricultural Research Center, Dokki, Giza 12618, Egypt; ahmed.erfan10000@gmail.com (A.M.E.); mohamedsamy_@yahoo.com (M.S.); osamamahana@yahoo.com (O.M.); dr_soadnasef@yahoo.com (S.A.N.)

**Keywords:** mule and pekin ducks, novel goose parvovirus, phylogenetic analysis, short beaks and dwarfism syndrome, *VP1* gene

## Abstract

**Simple Summary:**

Duck short beak and dwarfism syndrome is an emerging infectious disease caused by a novel goose parvovirus that has been detected in Europe and China since 1974. Low performance, slow growth and deaths of young ducklings were the main characteristics of the disease. To the best of our knowledge, such syndrome has not been recorded in Egypt, but since 2019, it was observed in some mule and pekin duck farms that resulted in drastic economic losses for waterfowl producers. Identification of the causative agent through viral and molecular detection of the causative virus was the aim of this study. Also, gene sequence of one of three viral protein genes which are responsible for the virulence was accomplished. The causative virus was isolated on primary cell culture, with partial gene sequence of viral *VP1* gene that indicated the viral clustering with Chinese novel goose parvoviruses that may help in new vaccine manufacturing and development of a more sensitive diagnostic assay. Future studies to evaluate potential protection of an available market vaccine against the novel virus will be useful.

**Abstract:**

Derzsy’s disease causes disastrous losses in domestic waterfowl farms. A genetically variant strain of Muscovy duck parvovirus (MDPV) and goose parvovirus (GPV) was named novel goose parvovirus (NGPV), which causes characteristic syndrome in young ducklings. The syndrome was clinically characterized by deformity in beaks and retarded growth, called short beaks and dwarfism syndrome (SBDS). Ten mule and pekin duck farms were investigated for parvovirus in three Egyptian provinces. Despite low recorded mortality rate (20%), morbidity rate was high (70%), but the economic losses were remarkable as a result of retarded growth and low performance. Isolation of NGPV was successful on primary cell culture of embryonated duck liver cells with a clear cytopathic effect. Partial gene sequence of the *VP1* gene showed high amino acids identity among isolated strains and close identity with Chinese strains of NGPV, and low identity with classic GPV and MDPV strains. To the best of our knowledge, this can be considered the first record of NGPV infections in Egypt.

## 1. Introduction

Parvovirus (a single-stranded DNA virus that belongs to family Parvoviridae) was thought to be the causative agent of outbreaks in young goslings and Muscovy ducklings within 1960, which was known as Derzsy’s disease [1,2]. In 1978, Derzsy’s disease was recommended to be termed as goose parvovirus (GPV), which caused great economic losses in the waterfowl industry. In 1989, a different strain of parvovirus was recorded in Muscovy ducks infected with similar symptoms to those of GPV [3,4,5], so, it was called Muscovy duck parvovirus (MDPV), with up to 85% nucleotide identity to GPV [6].

Both GPV and MDPV usually show 70–100% morbidity and mortality during the first 3 to 4 weeks of age [7,8]. The common clinical signs were growth retardation and watery diarrhea; then, within a few days, drastic mortalities took place [8,9]. According to the International Committee on Taxonomy of Viruses (ICTV), and due to similar evolutionary origins and genetic characters, GPV and MDPV were classified into the genus Dependoparvovirus, related to the subfamily Parvovirinae [10]. Both GPV and MDPV are antigenically related to each other as they possess about 85% protein sequence homology [11,12]. Novel goose parvovirus (NGPV) was firstly reported in 1970 in French mule duck flocks which showed growth retardation, beak atrophy and protruding tongue, and the disease was called short beak and dwarfism syndrome (SBDS) [13]. Thereafter, mortalities were usually recorded due to the inability of diseased duckling to eat or drink [14]. Muscovy and mule ducks could be affected with similar symptoms but mortality rate differs according to the age and immune status of the host [13]. Short beak and dwarfism syndrome in young ducklings was reported by the authors of Reference [15] in Taiwan, with 86% to 100% morbidity and mortality rates among different types of ducks during 1989 and 1990. Based on virus isolation and genetic sequence, NGPV was reported to cause SBDS in mule and pekin ducklings in China [14,16,17]. Recently, NGPV was associated with feather shedding either in single NGPV infection or mixed with duck circovirus in cherry valley ducks in China [18].

Earlier studies reported the possible vertical transmission of classic GPV in geese [19] and for MDPV [20]. Similarly, vertical transmission of NGPV from breeder cherry valley, pekin and mule ducks to their offspring was evidenced [17,21,22].

The genome analysis of GPV and MDPV indicated two open reading frames (ORFs), from which the right ORF encodes for three viral proteins (VP1, VP2 and VP3), while the left ORF encodes for non-structural (NS) proteins (NS1 and NS2) [5,11]. Viral proteins (VPs) are capsid proteins that are responsible for viral pathogenicity, antigenicity and attachment; therefore, VP gene sequencing is important for genetic characterization of newly emerged GPV [14,20,23,24]. Phylogenetic analysis of partial VP1 and VP3 sequences revealed that GPV strains from diseased cherry valley pekin and mule ducks with SBDS belong to the West-European lineage of GPV [13,17].

Egypt is considered as an important rest area for millions of migrating birds that migrate across Africa, Europe and Asia. Such birds usually carry pools of microbial agents that can easily be transmitted through contamination of the environment of the contact birds in the resting area. Different avian influenza strains were transmitted to the Egyptian birds through migratory birds [25].

Since 2019, some Egyptian duck farms have shown outbreaks marked by retarded growth, with some flocks suffering from short beaks and protruded tongues in mule and pekin duckling flocks. As far as we know, limited information is available regarding the NGPV and SBDS in Egypt. Therefore, molecular detection, isolation of causative virus from obtained samples from different diseased duck flocks showing high morbidity and/or mortality and genomic sequence analysis of these isolates were carried out.

## 2. Materials and Methods

### 2.1. Ethics Statement

Ten duck flocks showing different morbidity and mortality rates in young ducklings from three provinces (Giza, Behira and Qaliobia) in Egypt during 2019–2020 were sampled. Sample collection was approved by the Animal Care and Biosafety Committee of the Animal Health Research Institute (AHRI 121119). The diseased ducks were euthanized through intravenous injection of xylazine–ketamine solution before sample collection.

### 2.2. Samples and Samples’ Preparation

Ducklings 3–4 weeks of age from diseased farms suffering from variable rates of mortalities and retarded growth were subjected to postmortem examination. Collected organs (liver, spleen and heart) from each flock were homogenized using Qiagen LT Tissue lyser, which were then subjected to three successive freeze–thaw cycles, then a centrifugation step was performed at 18,000 rpm for 10 min to get the supernatant.

### 2.3. Polymerase Chain Reaction (PCR)

#### 2.3.1. Viral DNA Purification

Viral DNA was purified from 200 µl of the tissue homogenate supernatant from each infected flock by the QIAamp DNA mini kit (Qiagen, Gmbh, Hilden, Germany) following the manufacturer’s recommendation.

#### 2.3.2. PCR Amplification

PCR reactions were performed in 25 μL reactions using EmeraldAmp Max PCR Master Mix (Takara, Japan) in a Biometra T3000 thermocycler (Biometra, Gmbh, Germany). PCR targeted a 593 bp fragment of the *VP1* gene using specific primers (forward: 5′-CCTGGCTATAAGTATCTTGG-3′; reverse: 5′-GTAGATGTGGTTGTTGTAGC-3′) [11]. The PCR assay started with a primary denaturation step at 95 °C for 5 min, followed by 35 cycles (95 °C for 30 s, 55 °C for 40 s, 72 °C for 45 s), and a final extension step at 72 °C for 10 min. PCR products were inoculated into 1.5% agarose, and a gene ruler 100 bp ladder (Fermentas, Thermo scientific, MA, USA) was used to determine PCR assay specificity. The gel photo was captured using a gel documentation system (Alpha Innotech, Biometra, Gmbh, Germany) and the obtained photos were analyzed using Automatic Image Capture Software (Cell bioscience, USA). Synthetic oligonucleotides fragment was synthesized by Bio Basic, Markham, Canada, to serve as a PCR positive control (based on NGPV representative strain JS1603, GenBank accession MF441226).

### 2.4. DNA Sequence

A QIAquick PCR Product extraction kit (Qiagen, Gmbh, Hilden, Germany) was used to purify the 593 bp PCR products of the positive samples. The BigDye Terminator V3.1 cycle sequencing kit (Applied Bio-systems, Life Technologies, Thermofisher, MA, USA) was used to perform sequence reaction. The sequence reaction was then purified using Centri-sep spin columns (Thermofisher, MA, USA). Sequence chromatograms were retrieved from a 3130 genetic analyzer (Applied Bio-systems, Life Technologies, Thermofisher, MA, USA). To determine sequence identity to GenBank published parvoviruses, a BLAST^®^ search (Basic Local Alignment Search Tool) was performed [26]. A MegAlign module of Lasergene DNAStar was used to determine sequence identities among analyzed strains [27]. A neighbor-joining phylogenetic tree was constructed using the maximum composite likelihood model with 1000 bootstrap in MEGA6 [28].

### 2.5. Virus Isolation

The tissue homogenate supernatants were subjected to filtration using 0.2 μm sterile filters, then inoculated onto Primary duck embryo liver (DEL) cell culture. Negative control cell cultures were prepared as previously described [21,29]. The cell cultures were incubated at 37 °C for 7 days and checked daily for cytopathic effect. Cells showing detachment and/or clumping were considered positive for CPE. For validation, cell lysate was obtained for PCR testing to confirm isolation.

## 3. Results

### 3.1. Clinical Picture and Postmortem Findings

**Figure 1:**
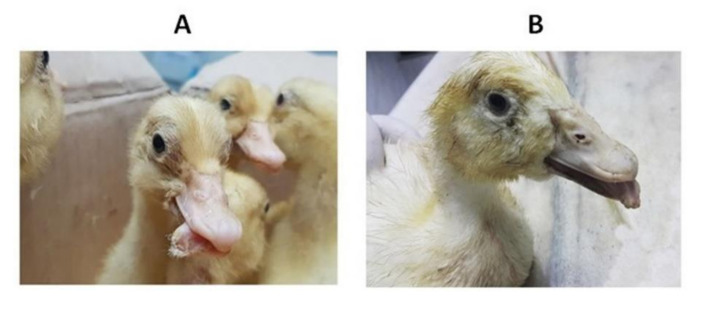
Characteristic signs of short beaks and protruded tongues in Pekin ducks (**A**) and mule ducks (**B**).

**Figure 2:**
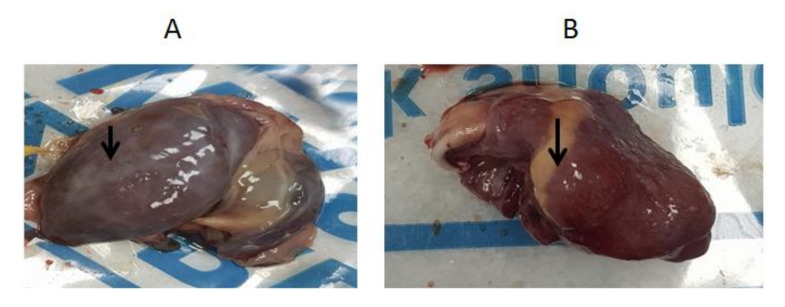
Observed PM (Postmortem) lesions: fibrinous perihepatitis (**A**) and hydropericardium (**B**).

Young duckling flocks of 3–4 weeks of age from three Egyptian provinces showed a variable morbidity rate of 70% and 30% mortalities. Suspected cases showed retarded growth associated with short beaks and protruded tongues in some diseased ducklings. Figure 1 shows clinical diseased pekin and mule ducklings with characteristic short beaks, protruded tongues and growth retardation. In ducks with clinical signs, ascites, hydropericardium and fibrinous perihepatitis were observed (Figure 2). As far as we can tell, no bacterial or viral disease agents other than NGPV can cause the revealed characteristic clinical signs (SBDS), since sampled birds were negative for Avian influenza virus, Duck hepatitis A virus, Salmonella, *E. coli*, *Pasteurella multocida* and *Mannheimia haemolytica*.

### 3.2. Identification and Isolation of the Causative Agent

**Figure 3:**
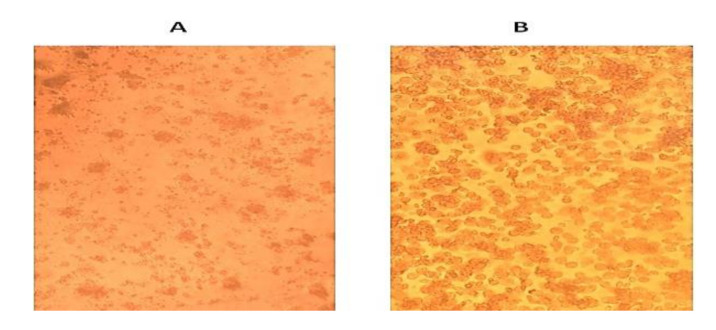
Viral inoculation onto duck embryo liver (DEL) cell culture: (**A**) Infected cells showing detachment and clumping, (**B**) non-infected DEL cell monolayer (negative control).

Four flocks showed positive amplification of a 593 bp fragment of the *VP1* gene for the RNA purified from tissue homogenates supernatants, where a valid result for the positive control was obtained. Isolation of the virus from tissue samples of diseased ducks with clinical signs of SBDS, which were found positive for parvovirus-specific DNA by PCR, was successful on primary duck embryo liver (DEL) cell culture. The virus showed detachment and clumping cytopathic effect with the four positive parvovirus strains after four days of incubation compared with the negative control normal confluent cell culture (Figure 3A,B). Positive PCR results for the harvested tissue culture fluids confirmed the successful isolation of the virus on DEL.

### 3.3. DNA Sequence and Phylogenetic Analysis

**Figure 4:**
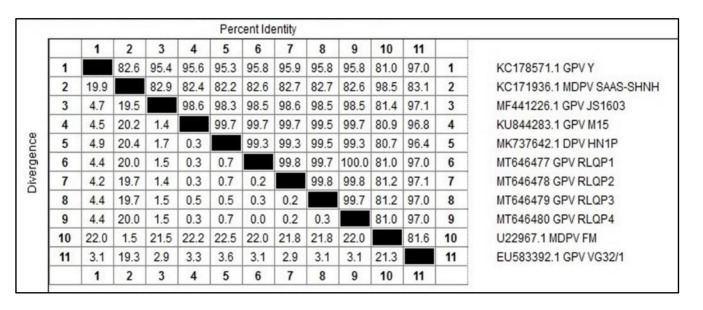
Nucleotide identities of the *VP1* gene for Egyptian isolates with selected references and vaccine strains.

**Figure 5:**
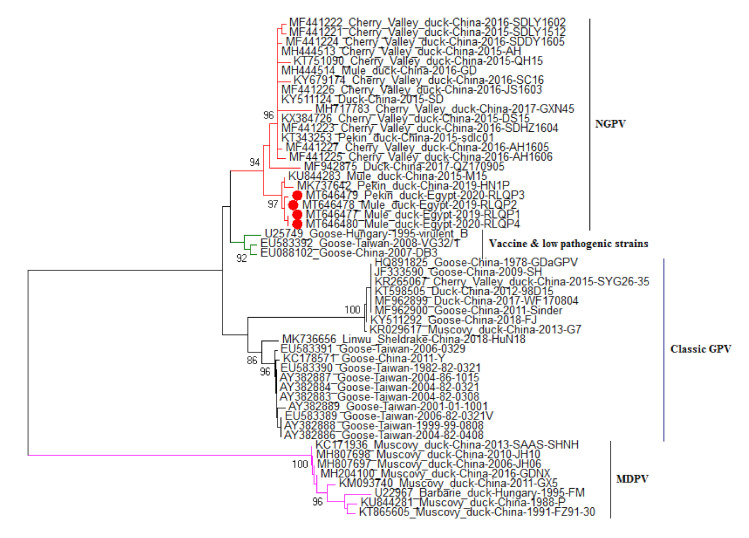
*VP1* gene Phylogenetic tree, with four Egyptian NGPVs (Novel Goose Parvovirus) strains (indicated by red dots) and other waterfowl parvovirus isolates with sequences available in the GenBank database. The phylogenetic tree was constructed using the neighbor-joining algorithm with 1000 bootstrap replicates. Bootstrap values are shown on the tree.

**Figure 6:**
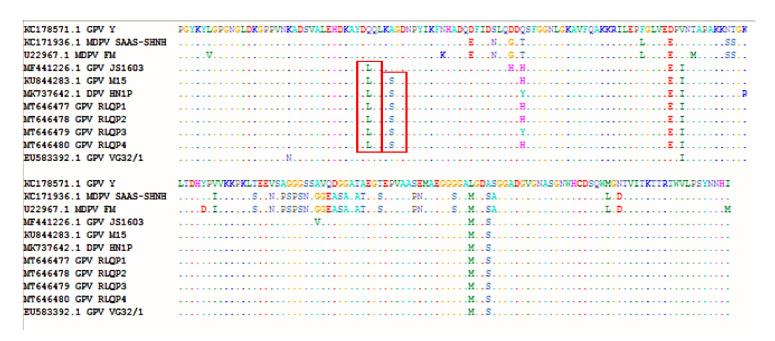
Amino acid alignment report created by Bioedit software showing close amino acids patterns of four RLQP (Reference laboratory for veterinary quality control on poultry production) strains with both Chinese NGPV strains (M15 and HN1P). Red bars are defining the Gln89Leu mutation that was present in NGPV strains, and the unique amino acid mutation (Ala93Ser) that was only recorded in the Egyptian NGPV strains and in two Chinese NGPV strains (M15 and HN1P).

DNA sequencing for 593 bp of *VP1* gene from 4 positive isolates were performed. Sequences were assigned GenBank accessions MT646477-MT646480. The sequence distances tool of the Lasergene DNAStar software revealed 99.7–100% identity among the current study strains. The sequenced strains were clustered within the novel goose parvovirus (NGPV) strains, where they showed 98.5–98.6% nucleotide sequence identity with NGPV representative strain JS1603 (GenBank accession MF441226) (Figure 4), however they showed 82.6–82.7% and 95.8–95.9% identities with the MDPV representative strain SAAS-SHNH (GenBank accession KC171936) and the classic goose parvovirus representative strain Y (GenBank accession KC178571), respectively. Selected strains for sequence analysis are shown in Figure 5. Although the phylogenetic analysis showed clear clustering of the four Egyptian strains within the NGPV cluster, they showed sub-clustering (99.3–99.7% identities) with two unique Chinese NGPV strains (GenBank accessions KU844283 and MK737642) that were recorded in 2015 and 2019, respectively (Figure 5). Both Chinese strains were responsible for SBDS outbreaks in Chinese mule ducks and pekin ducks, respectively. As shown in Figure 6, one amino acid mutation (Gln89Leu) was shared by the other NGPV strains and the Egyptian NGPV strains, apart from the classic GPV and MDPV strains. However, a unique amino acid mutation (Ala93Ser) was only recorded in the Egyptian NGPV strains and in two Chinese NGPV strains (KU844283 and MK737642). Unlike the common NGPV strains, two common amino acid mutations (Asp114His and Ala180Val) were not recorded in the Egyptian group. Also, the amino acid alignment (Figure 6) showed three mutations that were shared by the Egyptian and other NGPV strains and the MDPV strains, apart from the classic GPV strains (Asp142Glu, Leu207Met and Ala210Ser). Among the Egyptian NGPV strains, the four strains were 100% identical on the amino acid level except for the Gln116Tyr mutation that was reported in the RLQP3 strain (that was related to the Chinese NGPV strain MK737642), however the other three Egyptian strains showed the common Gln116His mutation.

## 4. Discussion

Classical GPV was characterized by retarded growth and severe mortalities in ducklings and goslings [7]. On the other hand, during the last ten years, SBDS dispersed in several countries, as it was first reported in France in the 1970s [13], followed by outbreaks in Taiwan (1989), Poland (1995) [2,30], Hungary (2009) [13] and mainland China (2014) [23].

During 2019–2020, the reference lab for veterinary quality control on poultry production (RLQP) examined ten mule and pekin duck farms from three provinces in Egypt (Giza, Behira and Qaliobia) that showed retarded growth and reduced performance, with characteristic clinical signs of short beaks and protruded tongues (SBDS), in four duckling farms with severe economic losses.

In the current study, and as the first record, the causative virus of SBDS was isolated and classified as Novel GPV (NGPV) and the partial gene sequence of *VP1* was analyzed. As mentioned before, the clinical findings of short beaks and protruded tongue for diseased ducklings in four duck farms were severely affecting their ability to eat and drink. Consequently, the diseased ducklings continued to exhibit poor growth and increased mortality. Although the observed mortalities for affected farms were not so high, the production performance of ducks that survived the infection was significantly reduced due to growth retardation [31]. Certain characteristics which justified the difference between NGPV and classical GPV were lower mortalities, resistance of mule and pekin ducks to GPV and the absence of pathological lesions in brain and kidneys in NGPV [32].

PCR is the preferred method for GPV detection from samples obtained from clinically affected ducks, as samples were usually successfully detected via PCR. The characteristic clinical signs of SBDS possessed a strong guide for presumptive diagnosis besides the confirmatory PCR result [33,34,35]. NGPV isolation was successful in duck embryo liver cell culture that showed detachment and clumping of liver cells, corresponding with Reference [21], who succeeded in isolation of NGPV in duck embryo liver cell. Also, successful isolation of NGPV was carried out by other researchers [34,36].

VP protein contains viral antigenic sites, so it plays an important role in the stimulation of protective response in the body against the virus [37]. In our study, there is no significant difference in sequence identity of the *VP1* gene among NGPV isolated from mule duck and those isolated from pekin duck, as they share 99.7–100% identity. The same results were found in a previous study when comparison was carried out between full *VP1* gene of NGPV from mule duck and Cherry Valley duck [35].

Phylogenetic analysis based on nucleotide sequence of the *VP1* gene was previously done to classify various strains of GPV and MDPV [14], and it showed clear clustering of the four Egyptian strains within the NGPV group and they were away from classic GPV and MDPV groups. Interestingly, it is the first study that reports NGPV infection in Egyptian ducks, and it might indicate that MDPV has not circulated in Egypt. However, more studies should be conducted since the reported cases for this infection are few. Furthermore, the Egyptian strains are closely related to two Chinese NGPV strains: the first one is the SBDSV M15 strain (Genbank accession No. KU844283) that was isolated from mule duck and caused 80% and 90% morbidity in 3-day-old mule and cherry valley ducklings respectively, upon experimental infection [23], and the second one is the HP1N strain (Genbank accession No. MK737642) that was isolated from pekin ducks that suffered from abnormal molting [31]. However, it was not known exactly how these variant strains were introduced into Egyptian duckling flocks. Many duck viruses were introduced into the Egyptian duck industry that were of Asian origin. Duck hepatitis A virus (DHAV) is one of the best examples for such introductions [38]. On the other hand, Egypt is regarded as one of the highest importing countries of one-day-old ducklings, especially from France, where SBDS was first reported in the 1970s. Such facts support the way of introduction of NGPV into the Egyptian duck industry, as vertical transmission of NGPV was previously reported [21]. The possible role of migratory birds for transmission of NGPV to Egypt should be kept in mind, as they were accused of transmission of different disease agents into Egypt, especially avian influenza viruses [39,40].

As previously reported by other researchers [16], the sequenced VP1 fragment was able to identify the four isolated strains as NGPV strains. Egyptian strains showed 98.5–98.6% nucleotide sequence identity with the NGPV representative strain, however they showed 82.6–82.7% and 95.8–95.9% identities with the MDPV and the classic GPV representative strains, respectively. The sequence analysis indicated that all NGPV isolates showed more homology among the NGPV cluster as compared to classical GPV and MDPV, but they were more genetically related to GPV than to MDPV [20,33,35]. NGPV was more readily recognized and neutralized by antisera from a GPV than by those from MDPV [14]. Egyptian strains showed 97–97.1% similarity with the VG32/1 vaccine strain (GenBank accession No. EU583392.1) which is related to classical GPV. On the other hand, the currently used Cevac Deparmune K vaccine is composed of 2 strains, the first one is the FM strain, GenBank accession No. U22967.1, which is related to MDPV, and this strain showed 81–81.2% identity with the current study strains. The second vaccine strain is the Derzsy LB strain (GenBank accession No. AY496900.1), which showed 95.7–96% identity when compared to the Egyptian strains (data not shown). Such similarity with the LB vaccine strain suggested its potential effectiveness against NGPV, but none of the infected farms in the current study were vaccinated with the CEVAC (Ceva Sante Animale vaccine) vaccine. Further studies are required to explore the effectiveness of currently available vaccines against NGPV or to develop a new NGPV vaccine.

VP1 protein is a surface-exposed protein that represents the major determinant of viral receptor binding and host specificity [41,42]. Several amino acid mutations in Egyptian strains related to NGPV were recorded in comparison to other classical GPV and MDPV strains, these mutations in VP1 protein may be responsible for virus evolution and subsequent adaptation and infection of mule and pekin ducks other than Muscovy ducks and geese. This hypothesis was previously suggested [14], but needs future experimental investigation. However, little information is known so far about the genetic determinants of GPV virulence and the genetic changes that are responsible for SBDS strains’ evolution. Finally, this study recommends the implementation of preventive strategies to control GPV outbreaks in Egyptian duck and goose farms and to increase awareness about the importance of GPV vaccination for breeder ducks and geese.

## 5. Conclusions

In summary, we reported the first record of NGPV cases in Egyptian duck flocks that is responsible for SBDS outbreaks. The isolation of the causative virus from ducks was successful on DEL cells. *VP1* gene PCR and sequencing were able to cluster the isolated virus within NGPV strains with close proximity to Chinese NGPV strains. In-time need for advanced surveillance of NGPV in Egypt must take place. Further studies are also needed to better understand the evolution and pathogenicity of viruses in different duck breeds and to determine the protective efficacy of the currently used vaccine against SBDS
